# Portrait of Professor J. D. White

**Published:** 1853-10

**Authors:** 


*Portrait of Professor J. D. White.*—We have received from Drs. W. W. Fouche, C. C. Williams and J. H. McQuillen, a most excellent lithograph likeness of Professor White, of the Philadelphia Dental College, gotten up in a manner highly creditable to the artists who executed it. The likeness is good, and we beg to return to the gentlemen who had the kindness to send it to us, our warmest thanks for so acceptable a present. Another, equally good, but of smaller size, was published in the April number of the Dental News Letter, which some good friend took from our table immediately after it was received. After waiting about three months, hoping it would be returned, we were compelled to write to the publishers for a duplicate copy. But for this circumstance we should have noticed it in the July number of the Journal.

